# Relationship between insulin resistance and thyroid cancer in Chinese euthyroid subjects without conditions affecting insulin resistance

**DOI:** 10.1186/s12902-022-00943-6

**Published:** 2022-03-07

**Authors:** Ning Xu, Haixia Liu, Yuan Wang, Yimiao Xue

**Affiliations:** Department of Endocrinology and Metabolism, The Second Hospital of Dalian Medical University, Dalian, 116027 P.R. China

**Keywords:** Thyroid nodule, Insulin resistance, Thyroid cancer

## Abstract

**Backgrounds:**

In recent years, many studies have shown that insulin resistance is related to the occurrence of thyroid cancer, but there are few reports on whether the two are related under the premise that thyroid function is normal and the metabolic components related to insulin resistance are excluded. This study aims to analyze the insulin resistance of patients with differentiated thyroid cancer after excluding the population with abnormal metabolic components, and to study the risk factors of thyroid cancer in this population.

**Methods:**

61 subjects diagnosed with differentiated thyroid carcinoma (DTC) formed the DTC group and 262 subjects with benign nodules formed the control group. Body mass index (BMI, kg/m2), waist circumference (WC), lipid profiles, and free T3 (FT3), free T4 (FT4), thyroid-stimulating hormone (TSH), thyroid peroxidase antibody (TPOAb), thyroid globulin antibody (TGAb), alanine transaminase (ALT), aspartate aminotransferase (AST), fasting plasma glucose (FPG), fasting serum insulin and homeostatic model assessment of insulin resistance (HOMA-IR) levels were measured.

**Results:**

Mean subjects age (*P* = 0.021), BMI (*P* = 0.049), WC (*P* = 0.01), serum insulin concentration (*P* = 0.006), and HOMA-IR level (*P* = 0.005) were significantly greater in the DTC group than in the control group. Multivariate binary logistic regression analysis identified advanced age (OR = 1.027 [1.003–1.051], *P* = 0.029) and an increased HOMA-IR level (OR = 1.572 [1.277–1.935], *P* < 0.001) as significant risk factors for thyroid cancer.

**Conclusions:**

IR may increase the risk of thyroid cancer development even in the absence of conditions affecting insulin resistance.

## Introduction

The incidence of thyroid cancer has continued to increase worldwide in recent decades, primarily due to the increased incidence of differentiated thyroid carcinomas (DTCs), and more specifically, papillary thyroid carcinomas (PTCs) [1]. Although the reasons behind this increase remain unclear, environmental factors have been studied as potential causative factors in recent years. For example, one study showed that obesity is closely associated with thyroid cancer [2]. Other studies have shown that there is significant correlation between DTC and insulin resistance [3]. Akker et al. determined that obesity and high homeostasis model assessment of insulin resistance (HOMA-IR) levels are more important in the development of DTC than IR-related polymorphisms [4]. However, additional associative data revealing the effect of IR on the development of thyroid cancer are lacking.

Body mass index (BMI) is a measure of weight relative to height (kg/m2) that indicates body fat composition, and the World Health Organization (WHO) recommends a median BMI in the range of 21–23 kg/m2 for optimal health among adults. For most individuals, the general indication is to maintain a BMI in the range of 18.5–24.9 kg/m2, given that many studies have demonstrated increased risks of comorbidities in individuals with BMIs ranging from 25.0–29.9 kg/m2 and moderate to severe risks of comorbidities in individuals with a BMI greater than 30 kg/m2 [5, 6]. Obesity and overweight are risk factors for cancer in 13 body parts [7]. Metabolic syndrome (MetS) is widespread around the world, it is a cluster of metabolic abnormalities that has been recognized as a risk factor for vascular events as well as an increased overall mortality [8]. Studies on MetS have strongly suggested a central role for IR. IR specifically has been reported to be a risk factor in breast and endometrial cancer, hepatocellular carcinoma, colorectal cancer [9–12]. Insulin-like growth factor-1 (IGF-1) pathway has been suggested to be associated with canceration and cancer progression [13].

Although some studies have investigated the effect of IR in the development of thyroid cancer [14], little information is available regarding their association between insulin resistance and thyroid cancer in euthyroid subjects without insulin resistance-related risk factors such as diabetes mellitus, a past history of cardiovascular disease, and hypertension. The incidence of thyroid cancer is expected to increase gradually from that in the obese population to that among individuals with impaired glucose tolerance through to that among individuals with early stage type 2 diabetes placed under diet control without pharmacological intervention [15]. The aim of this study was to investigate any relation between IR and thyroid cancer in subjects without IR-related risk factors. A secondary objective was to explore factors associated with thyroid cancer in this population.

## Materials and methods

### Study design

The study was a cross-sectional study in subjects with euthyroid nodular diseases. We collected data for 398 subjects with thyroid nodules who underwent cytological biopsy of the thyroid gland and the biopsy results were clear (Bethesda II + V + VI) at the Second Hospital of Dalian Medical University between February 2018 and March 2020. In the study population, we used the Thyroid Imaging Reporting and Data System (TI-RADS) by the American College of Radiology (ACR) for assessing nodule risks for decision-making on biopsy, ultrasound judged TI-RADS3 nodules larger than 1 cm, and TI-RADS4 and above nodules larger than 5 mm were considered for puncture. The study was approved by the medical ethics committee of our hospital, and all participants provided informed consent. Of the 398 subjects with thyroid nodules who underwent cytological biopsy of the thyroid gland at our hospital, 23 subjects were excluded due to the presence of hypertension; 5 subjects were excluded due to a past history of polycystic ovary syndrome (PCOS) and a family history of diabetes; 9 subjects were excluded due to a past history of cardiovascular disease or having electrocardiographic abnormalities suggestive of coronary artery disease; 3 subjects were excluded due to use of thyroid medication; and 5 subjects were excluded due to a diagnosis of infection; subjects with the symptoms of diabetes and a fasting plasma glucose 7.0 mmol/L or greater were excluded from the study. If subjects had no symptoms and a FPG was 7.0 mmol/L or greater, then they had a second measurement, and if the FPG again was 7.0 mmol/L or greater, then they were excluded from the study. Thus 30 diabetic subjects were excluded, and all included subjects had a FPG less than 7.0 mmol/L. The final study population was divided into two groups according to the diagnosis of DTC or benign histopathology after biopsy. Biopsy results was classified by Bethesda classification, there were no uncertain biopsy results (Bethesda I + III + IV), The diagnosis category of DTC in this study is Bethesda V + VI nodules, The diagnosis category of benign histopathology in this study is Bethesda II nodules. Thus, the study included 323 subjects, and of these, 61 subjects were diagnosed with DTC after biopsy (DTC group) and 262 subjects were diagnosed with a benign tumor after biopsy (control group). All subjects had normal TSH (reference range, 0.51–4.94 mIU/mL), FT3 (reference range, 3.5–6.5 pmol/L), and FT4 (reference range, 12–22 pmol/L) levels.

### Anthropometric measurements

Anthropometric measurements were taken by physicians or trained nurses with individuals wearing light clothing and no shoes. Weight was measured to the nearest 0.1 kg using mechanical scales. Height was measured to the nearest 0.1 cm using a freestanding automatic stadiometer. Each subject’s BMI was calculated by dividing the body weight (kg) by height squared (m2). Waist circumference (WC) was measured in centimeters using a folding tape on bare skin between the 10th rib and the iliac crest.

### Laboratory assessments

Venous samples were drawn after a minimum fasting period of 12 h. All samples were collected between 06:00 and 07:00 h. The concentrations of serum glucose, total cholesterol, triglyceride (TG), high-density lipoprotein (HDL-C), low-density lipoprotein (LDL-C), alanine transaminase (ALT) and aspartate aminotransferase (AST) were measured by spectrophotometry using an Advia 2400 Clinical Chemistry System (Siemens, USA). Insulin, FT3, FT4, TSH, thyroid peroxidase antibody (TPOAb), and thyroid globulin antibody (TGAb) were determined by a chemiluminescent immunometric assay using a Centaur XP chemiluminescent analyzer (Siemens, USA). The HOMA was used as a measure of insulin sensitivity using the equation = fasting insulin (mU/L) × glucose (mmol/L)/22.5. IR was defined by a HOMA value > 2.7 as suggested by Matthews et al. [16].

### Assessment of cardiovascular disease

All subjects were questioned regarding medication usage and having a history of cardiovascular diseases (coronary artery disease or cerebrovascular disease).

### Statistical analysis

The Kolmogorov–Smirnov test was used to analyze the distribution pattern. Continuous variables were presented by mean ± standard deviation. Categorical variables were presented as numbers and percentage. The Chi Square test was used for comparisons of categorical variables. A multivariate binary logistic regression analysis was used for the assessment of risk factors for the development of DTC. Pearson correlation analysis is used to detect the relationship between age and HOMA-IR. P < 0.05 was considered statistically significant. Statistical analysis was performed with SPSS 17.0 statistical software using the production facility evaluation mode.

## Results

The clinical and laboratory data of the subjects in both groups are provided in Table 1. A total of 323 patients (61 in the DTC group and 262 in the control group) were included in the final analysis. No significant differences in gender, blood pressure, fasting plasma glucose (FPG), HDL-C, LDL-C, total cholesterol, FT3, FT4, TG, TPOAb, TGAb, TSH, ALT, and AST levels were observed between the two groups. The mean age of subjects in the DTC group was greater than that in the control group (AgeDTC = 54.9 ± 13.0 years, Agecontrol = 50.3 ± 14.0 years, *P* = 0.021), and subjects in the DTC group also had a greater mean BMI (BMIDTC = 25.6 ± 2.6 kg/m2, BMIcontrol = 24.9 ± 2.6 kg/m2, *P* = 0.049) and WC (WCDTC = 89.4 ± 10.1 cm, WCcontrol = 86.1 ± 8.4 cm, *P* = 0.01). Moreover, the mean HOMA-IR (HOMA-IRDTC = 5.4 ± 6.9, HOMA-IRcontrol = 2.8 ± 1.3, *P* = 0.005) and insulin (insulinDTC = 22.7 ± 30.0 μU/mL, insulincontrol = 11.8 ± 5.4 μU/mL, *P* = 0.006) levels were higher in the DTC group than in the control group.

**Table 1:** Baseline characteristics of the DTC group and control group

**Characteristics**	**DTC**	**Control**	***P* value**
	**(*n* = 61)**	**(*n *= 262)**	
Gender (female/male)	44 / 17^1^	182 / 80^1^	0.683
Age (years)	54.9 ± 13.0^1^	50.3 ± 14.0^1^	0.021
BMI (kg/m2)	25.6 ± 2.6^1^	24.9 ± 2.6^1^	0.049
WC (cm)	89.4 ± 10.1^1^	86.1 ± 8.4^1^	0.01
Systolic pressure (mmHg)	128.1 ± 10.3^1^	125.4 ± 10.1^1^	0.054
Diastolic pressure(mmHg)	80.5 ± 8.3^1^	79.1 ± 7.9^1^	0.189
FT3 (pmol/L)	4.8 ± 0.7^1^	4.7 ± 0.6^1^	0.410
FT4 (pmol/L)	16.0 ± 2.6^1^	16.4 ± 2.6^1^	0.196
TSH (mIU/mL)	2.3 ± 1.2^1^	2.1 ± 1.3^1^	0.598
FPG (mmol/l)	5.3 ± 0.3^1^	5.3 ± 0.4^1^	0.922
HDL-C (mg/dl)	1.3 ± 0.3^1^	1.4 ± 0.5^1^	0.337
LDL-C(mg/dl)	2.9 ± 0.8^1^	2.9 ± 1.3^1^	0.874
Total cholesterol (mmol/L)	5.6 ± 4.2^1^	4.9 ± 1.1^1^	0.052
TG (mmol/L)	1.6 ± 1.0^1^	1.7 ± 2.5^1^	0.735
TPOAb (U/mL)	68.8 ± 87.6^1^	76.8 ± 121.9^1^	0.628
TGAb (U/mL)	78.0 ± 159.6^1^	81.3 ± 137.4^1^	0.761
ALT (U/L)	24.9 ± 15.9^1^	23.2 ± 12.7^1^	0.364
AST (U/L)	22.3 ± 9.3^1^	23.5 ± 8.0^1^	0.296
Insulin (pmol/l)	22.7 ± 30.0^1^	11.8 ± 5.4^1^	0.006
HOMA-IR (µU/mL)	5.4 ± 6.9^1^	2.8 ± 1.3^1^	0.005

^1^Numbers are mean ±SD and n for gender

In the final multivariate analysis model, predictors of DTC were selected based on both their clinical and statistical significance [17]. A backward elimination method was applied to these results to select the most important predictor variables. The results of multivariate binary logistic regression analysis in Table 2 revealed that the risk factors with a significant effect on the development of cancer in DTC subjects were advanced age (odds ratio [OR] = 1.027 [1.003–1.051], *P* = 0.029) and an elevated HOMA-IR level (OR = 1.572 [1.277–1.935], *P* < 0.001). Whereas elevated BMI (OR = 1.089[0.971–1.221], *P* = 0.147), gender (OR = 1.479[0.723–3.027], *P* = 0.284), TPOAb (OR = 0.999[0.996–1.002], *P *= 0.554) and TGAb (OR = 1.001[0.999–1.003], *P *= 0.459) were not significantly associated with the risk of cancer in DTC. Also, we have checked the relationship between age and HOMA-IR in entire cohort, The tested of Pearson correlations of Age and HOMA-IR showed that the index were tested not correlation (*P* = 0.682), proved that HOMA-IR is actually related to DTC diagnosis (rather than another marker related to the age).

**Table 2:** Multivariate binary logistic regression analysis for the assessment of risk factors for the development of DTC

	**B**^2^	**S.E**^2^	**P value**	**OR (95%)**^2,3^
Age (years)	0.026	0.012	0.029	1.027(1.003–1.051)^1^
BMI (kg/m2)	0.137	0.058	0.147	1.089 (0.971–1.221)^1^
Gender	0.421	0.369	0.284	1.479(0.723–3.027)^1^
TPOAb (U/mL)	< 0.001	0.002	0.554	0.999(0.996–1.002)^1^
TGAb (U/mL)	0.001	0.001	0.459	1.001(0.999–1.003)^1^
HOMA-IR(µU/mL)	0.444	0.107	< 0.001	1.572(1.277–1.935)^1^

^1^Baseline values in tables represent statistical means and confidence intervals

^2^B: unstandardized regression coefficients; S.E: standard error; OR: odds ratio

^3^OR were computed with the use of multivariate binary logistic regression model

## Discussion

Our results demonstrated that there is an association between IR and the pathogenesis of DTC in our euthyroid patients without insulin resistance-related risk factors. The age, BMI, WC, insulin, and HOMA-IR levels of subjects with DTC were significantly higher than those of subjects in the control group. IR leads to compensatory increased insulin secretion and is characterized by decreased tissue sensitivity to the action of insulin [18]. Patients with IR are at increased risk for cardiovascular diseases and type 2 diabetes. Moreover, IR is a state of impaired glucose metabolism. It results from reduced sensitivity of the target tissues (muscles, adipose tissue, liver) to insulin and may lead to the development of type 2 diabetes. IR is also linked to other metabolic abnormalities, such as obesity, type 2 diabetes, and metabolic syndrome [14]. Furthermore, abnormal thyroid function may lead to metabolic abnormalities and even IR. For these reasons, we excluded patients with a history of cardiovascular disease or diabetes and selected euthyroid individuals in order to decrease interferring factors. One study concluded that subjects with IR have larger thyroid volumes and thyroid nodules, it showed that IR may induce increased thyroid proliferation and nodule volume and nodule formation [19]. Moreover, increased prevalence of IR has been observed in subjects with DTC. Sahin et al. demonstrated that insulin, HOMA-IR, and LDL-C levels are significantly higher in subjects with DTC than in healthy controls [20]. Consistent with the study results, our study showed that levels of insulin and HOMA-IR in the DTC group were higher than those in the control group. Additional research has suggested a pivotal role for IR in the distribution, construction, and density of thyroid nodular vascularization, which might contribute to the growth and progression of nodules [21]. In our study, the mean HOMA-IR (*P* = 0.005) and insulin (*P* = 0.006) levels were higher in the DTC group than in the control group, and multivariate binary logistic regression analysis showed the increased HOMA-IR level is associated with the development of thyroid cancer. However, inconsistent with our results, Fevzi et al. found that IR is not more prevalent in subjects with thyroid cancer than in those with benign thyroid nodules and the difference between subgroups based on tumor size was statistically insignificant [22], This discrepancy may be related to the limited sizes of their groups, and more data are needed to confirm the accuracy of their results.

MetS involves a cluster of risk factors including increased blood pressure, abdominal obesity, lipid abnormalities, and impaired glucose metabolism. It has been associated with elevated risks of several common adult cancers such as thyroid cancer, and the data suggest that among typical metabolic abnormalities such as MetS, IR and MetS-related components may contribute to increased thyroid volume and nodule prevalence, even leading to tumor formation [19]. There are several possible mechanisms. The insulin-like growth factors (IGFs) play a major role in a variety of human malignancies as potent mitogenic and anti-apoptotic factors [23], and one study reported that serum IGF-1 concentrations may be positively associated with risk of DTC [24]. Both IGF-1 and IGF-2 are produced locally in thyroid tumors, and several studies found that IGF-1 is positively correlated with thyroid volume, independently of age and body size, and studies have also shown that growth hormone (GH) / IGF-1 axis is associated with thyroid volume growth [25, 26].Whereas high serum IGF-1 levels are also related to thyroid nodule formation in men, they are related to decreased serum TSH levels in women [27]. Some findings have supported the hypothesis that both IGF-1 receptor (IGF-1R) and insulin receptor play a role in the differentiation of thyroid progenitor/stem cells and that thyroid cell differentiation is associated with the downregulation of insulin receptor and IGF-1R and a reduction in the ratio of insulin receptor A to insulin receptor B [28]. Hyperglycemia is associated with increased levels of proinflammatory factors that contribute to tumor neoangiogenesis, such as tumor necrosis factor (TNF)-α, transforming growth factor (TGF)-α, TGF-β, interleukin (IL)-8, vascular endothelial growth factor (VEGF)-A, and fibroblast growth factor 2 [29]. In accordance with these findings, insulin receptor expression was found to be upregulated in hypofunctioning benign thyroid adenomas that have lost a differentiated function such as iodine uptake, indicating that overexpression of insulin receptor occurs early in thyroid tumorigenesis [30]. Obesity is a major risk factor for numerous cancers such as endometrial, colon, rectal, kidney, pancreatic, and thyroid cancer [10]. A meta-analysis shows that high BMI increases the risk of thyroid cancer [31]. A recent, retrospectively studies showed that obesity does not modify the risk of differentiated thyroid cancer in a cytological series of thyroid nodules [30]. Our study showed that subjects in the DTC group had a greater mean BMI (*P* = 0.049) than those in the control group. Whereas elevated BMI was not significantly associated with the risk of cancer in DTC.

In a cross-sectional study measuring both exposure and disease conditions, it is difficult to determine the cause and effect, and this is a major weakness. Therefore, by including the multivariate binary logistic regression analysis,our results could show that IR may be a risk factor for thyroid cancer. In our research, fasting blood glucose >  = 7.0 mmol/L was used as the single diagnostic criteria may under-diagnose diabetes, and therefore the cohort may include a population with early-stage diabetes and intermittent hyperglycemia. Another limitation of our study is that many chronic diseases are likely to result in relative deterioration or paracmasis, and thus, a cross-sectional study may treat patients in paracmasis as healthy during the research. Finally, our entire study population was Chinese (100%).

In conclusion, we determined that the incidence of IR among subjects with DTC was higher than that among subjects with a benign thyroid nodule. Additionally, the high age and elevated HOMA-IR were identified as potential risk factors for the development of thyroid cancer. Thus, IR may be a risk factor for thyroid cancer. IR subjects with thyroid nodules should be followed up closely, and if necessary, thyroid cytology biopsy should be performed to facilitate the identification and immediate treatment of cancerous nodules to improve the prognosis of these subjects.

## Data Availability

The data that support the findings of this study are available from the corresponding author upon reasonable request.

## Abbreviations

IR: Insulin resistance
DTC: Differentiated thyroid carcinoma
BMI: Body mass index
WC: Waist circumference
FT3: Free T3
FT4: Free T4
TSH: Thyroid-stimulating hormone
TPOAb: Thyroid peroxidase antibody
TGAb: Thyroid globulin antibody
ALT: Alanine transaminase
AST: Aspartate aminotransferase
FPG: Fasting plasma glucose
HOMA-IR: Homeostatic model assessment of insulin resistance
DTCs: Differentiated thyroid carcinomas
PTCs: Papillary thyroid carcinomas
WHO: World Health Organization
MetS: Metabolic syndrome
IGF-1: Insulin-like growth factor-1
PCOS: Polycystic ovary syndrome
TG: Triglyceride
HDL-C: High-density lipoprotein
LDL-C: Low-density lipoprotein
IGFs: Insulin-like growth factors
GH: Growth hormone
IGF-1R: Insulin-like growth factor-1 receptor
TNF: Tumor necrosis factor
TGF: Transforming growth factor
IL: Interleukin
VEGF: Vascular endothelial growth factor
